# Visual search behaviors of association football referees during assessment of foul play situations

**DOI:** 10.1186/s41235-016-0013-8

**Published:** 2016-10-31

**Authors:** Jochim Spitz, Koen Put, Johan Wagemans, A. Mark Williams, Werner F. Helsen

**Affiliations:** 1 Department of Kinesiology, Laboratory of Perception and Performance, Movement Control and Neuroplasticity Research Group, University of Leuven (KU Leuven), Tervuursevest 101, (box 1501), B – 3001 Leuven, Belgium; 2 Department of Brain & Cognition, Laboratory of Experimental Psychology, University of Leuven (KU Leuven), Tiensestraat 102, (box 3711), B – 3000 Leuven, Belgium; 3 Centre for Cognitive Neuroscience, College of Health and Life Sciences, Brunel University, London, UK

**Keywords:** Expertise, Sports officials, Decision making, Eye-tracking

## Abstract

It is well reported that expert athletes have refined perceptual-cognitive skills and fixate on more informative areas during representative tasks. These perceptual-cognitive skills are also crucial to performance within the domain of sports officials. We examined the visual scan patterns of elite and sub-elite association football referees while assessing foul play situations. These foul play situations (open play and corner kick situations) were presented on a Tobii T120 Eye Tracking monitor. The elite referees made more accurate decisions and differences in their visual search behaviors were observed. For the open play situations, referees in the elite group spent significantly more time fixating the most informative area of the attacking player (contact zone) and less time fixating the body part that was not involved in the infringement (non-contact zone). Furthermore, the average total fixation time in the contact zone and non-contact zone tended to differ between the elite and sub-elite referees in corner kick situations. In conclusion, elite level referees have learned to discern relevant from less-relevant information in the same way as expert athletes. Findings have implications for the development of perceptual training programs for sport officials.

**Electronic supplementary material:**

The online version of this article (doi:10.1186/s41235-016-0013-8) contains supplementary material, which is available to authorized users.

## Significance

For our understanding of basic processes like attention control, eye movement control, and selection of information for perceptual and cognitive processing, it is important to extend research beyond contrived laboratory conditions.

Officials in team sports are a specific population. They are responsible for interpreting and enforcing the laws of the game on the field of play, thereby protecting the players from potential injuries. Apart from the maintenance of “fair play”, the decisions of referees can affect the outcome of a game significantly. Perceptual and cognitive skills are required to make sure that the decision-making process results in accurate, consistent, and uniform decisions. Researchers have demonstrated that science-based, off-field perceptual-cognitive training protocols can increase accuracy of the decision-making process.

Characterizing the underlying mechanisms of expertise is fundamental to guiding the training process of (future) experts. In this study, we provide evidence for differences in the perceptual-cognitive processes of expert officials, enriching our insights into the interplay between bottom-up and top-down selection of information. The rich, complex situations which officials encounter are representative of many of the challenging tasks that experts carry out in their professional lives and findings have implications for the development of perceptual-cognitive expertise across domains.

## Background

To achieve expert performance in many sports, well-developed motor skills are required and athletes have to adapt their movements continuously to meet the ever-changing demands of the performance environment. Researchers have already examined the basic principles of motor control needed to produce biomechanically efficient and appropriate movements (De Witt & Hinrichs, 2012; Worthington, King, & Ranson, 2013). Perception often precedes appropriate action and over recent years more knowledge has been gathered about the contribution of perceptual-cognitive skills to expert performance (Williams & Ericsson, 2005). Scientists have demonstrated that experts are more accurate and faster in sport-specific tests of anticipation compared with novice counterparts (Mann, Williams, Ward, & Janelle, 2007; North, Ward, Ericsson, & Williams, 2011; Roca, Ford, McRobert, & Williams, 2011; Williams, Ward, Ward, & Smeeton, 2008). According to Ericsson and Kintsch (1995), expert performers develop long-term working memory (LTWM) skills as a result of accumulated deliberate practice, which allow them to retrieve critical and task-relevant information from long-term memory in an efficient way. These elaborated retrieval skills ensure control over strategic aspects of performance execution.

For sports officials, the requirement to process information in an accurate and adequate manner may be deemed even more important given there is no need to perform subsequent controlled and coordinated actions (MacMahon et al., 2014). Consequently, researchers have shown clear expertise effects in referee-specific decision-making tasks across various team sports, such as ice hockey (Hancock & Ste-Marie, 2013), rugby (Mascarenhas, Collins, Mortimer, & Morris, 2005), and association football (Catteeuw, Helsen, Gilis, Van Roie, & Wagemans, 2009; Gilis, Helsen, Catteeuw, & Wagemans, 2008).

An efficient and effective use of vision is definitely needed to scan the environment and to process relevant information prior to making decisions (Plessner & Haar, 2006). Expert and novice athletes are not characterized by differences in tests of basic visual function, such as visual acuity or light sensitivity (Helsen & Starkes, 1999a), but rather they differ in their visual search behavior (for a review, see Vickers, 2007). Visual search behavior is characterized by eye movements and fixations, which ensure that visual input is available to the cognitive system. Fixating a particular location is an observable, behavioral manifestation of the allocation of attention (Henderson, 2013). In time-constrained decision-making tasks, expert sport performers tend to use more pertinent visual search strategies, generally involving fewer but longer fixations. These longer fixations allow more time for information extraction from the display (Mann et al., 2007). Moreover, as a result of “perceptual automatization”, expert sport performers fixate on more informative areas of the display (Williams, Ward, Knowles, & Smeeton, 2002). Roca, Ford, McRobert, and Williams (2013a) reported that less skilled soccer players are guilty of “ball watching”, which is not an optimal source of information prior to deciding on an appropriate course of action. Skilled players, on the contrary, spend significantly more time fixating the informative areas of the display (i.e., the opponents and the areas of free space). The perceived visual information is encoded and given meaning during subsequent steps of information processing, resulting in a final behavioral response (Lachman, Lachman, & Butterfield, 2015). The visual search strategy has been reported to be specific for the domain of expertise and the task characteristics (North, Williams, Hodges, Ward, & Ericsson, 2009; Roca et al., 2011; Roca et al., 2013a; Vaeyens, Lenoir, Williams, & Philippaerts, 2007).

Paying attention to the relevant information at the right time and integrating this information with existing knowledge is one of the key components in refereeing (MacMahon et al., 2014). Thus far, only two published reports have investigated visual search strategy and its relative contribution in making decisions within the domain of refereeing. Catteeuw et al. (2009) first studied the decision-making process of international and national assistant referees using an eye-tracking system. The assistant referees of international level did not differ in the number and duration of fixations compared with the national expert assistant referees while assessing offside situations. Furthermore, no differences were present when analyzing the time spent fixating specific informative areas of the display (i.e., the passer and the offside line) at the moment of the pass. A second study showed that higher- and lower-level referees in ice hockey differ in their accuracy scores for a specific decision-making test but no differences in their visual search rate were found (Hancock & Ste-Marie, 2013). It was concluded that more accurate decisions within expert groups of referees are attributed to how information is interpreted (categorization: “seeing as”) rather than how it is gathered by the visual system (perception: “seeing”). However, this conclusion is speculative as these authors did not take into account the fixation location and, therefore, we cannot rule out that experts are able to extract more relevant decision-making information.

In light of the limited number of studies conducted within the field of refereeing and the importance of the decision-making processes in that domain, we examined the visual search strategies and decision-making processes of elite and sub-elite association football referees. We wanted to enhance understanding of the way association football referees make decisions. Tracing and understanding the underlying mechanisms of expertise is fundamental to guide and improve the decision-making process (Ericsson & Smith, 1991). For example, researchers have indicated that the perceptual-cognitive skills of referees and players can be improved via diverse on- and off-field training tools. During these sport-specific training programs, the focus should be on improving specific aspects of performance (Put, Wagemans, Spitz, Williams, & Helsen, 2015; Schweizer, Plessner, Kahlert, & Brand, 2011). Also, in other professional settings, such as airport baggage screening, medical screening, and law enforcement, findings from (basic) visual search studies have had important applications for training and performance enhancement (Evans, Birdwell, & Wolfe, 2013; Helsen & Starkes, 1999b; Vickers & Lewinski, 2012; Wolfe, Brunelli, Rubinstein, & Horowitz, 2013).

We examined the visual scan patterns of elite and sub-elite association football referees while assessing foul play situations (i.e., open play and corner kick situations) filmed from an in-game perspective. According to previous research, we did not expect differences in the visual search rates employed by referees differing in expertise level (Catteeuw et al., 2009; Hancock & Ste-Marie, 2013). However, referees must be able to identify the most informative areas of the display to see whether there is actually contact between players which could result in a foul or not. They have to direct their attention appropriately and interpret information from these areas efficiently and effectively. Given the association between the location of visual fixations and decision-making skills in various sports and the fact that the fixation locations have never been investigated during a foul play assessment task, we did expect differences between both groups of referees for fixation location. We hypothesized that referees in the elite group would spend significantly more time fixating informative areas of the visual display compared with sub-elite referees during the most crucial time intervals of the situations. More specifically, for the open play situations, we expected that elite referees would spend more time fixating the contact zones of both the attacking and defending player (i.e., the body part which was involved in the possible infringement). For the corner kick situations, we expected that the elite referees would spend more time fixating the contact zones (i.e., the area of the display which contained the two players who interacted for a possible infringement). Furthermore, we studied the decision-making accuracy of referees during the assessment of foul play situations (i.e., open play and corner kick situations). In line with previous research (Catteeuw et al., 2009; Hancock & Ste-Marie, 2013), we hypothesized that the elite group of referees would outperform the sub-elite group of referees with respect to the accuracy scores for the assessment of both types of situations. For the situations in which an incorrect decision was given, we determined for both groups how visual attention was allocated and what could be the cause of the error. In order to make a correct decision, referees first need to select and process the most informative areas of the visual display. A total visual fixation time of 1000 ms is considered a significant allocation of visual attention (Hillstrom, 2000; Nodine, Mello-Thoms, Kundel, & Weinstein, 2002). Second, the perceived situation must be categorized according to the Laws of the Game, integrating the available information into a final decision (Plessner & Haar, 2006). Errors could thus occur at two levels of the decision-making process: 1) a perception error if the referee failed to focus for a significant amount of time (1000 ms) in the contact zones; 2) a categorization error if the contact zone was perceived thoroughly but the referee failed to categorize the situation correctly.

## Methods

### Participants

Thirty-nine referees from Belgium were recruited according to their competitive level. A first group consisted of elite referees (n = 20, mean age 33.1 years, standard error (SE) = 1.4, range 24–45), all actively involved in the first and second highest division of professional football. The second group of sub-elite referees (n = 19, mean age 32.8 years, SE = 1.8, range 23–50) was active at lower competitive levels and had no refereeing experience at the professional level (Table 1). Participants provided written informed consent and the study was approved by the local University ethics committee (G-201504218).

**Table 1:** Mean number of years (and standard errors) of refereeing experience and experience at professional level for the elite and sub-elite group of referees

Group	Refereeing experience	Experience at professional level
Elite	16.3 (1.3)	9.3 (1.2)
Sub-elite	12.1 (1.3)	-

### Test film

Video clips of foul play situations were filmed from the first-person perspective of an additional assistant referee (i.e., filmed from the position next to the left goal post) with a Sony PMW-F55 4 K digital cinema camera. A foul is an unfair act by a player, deemed by the referee to contravene the Laws of the Game of the Fédération Internationale de Football Association (FIFA). A list of specific offences that can be categorized as a foul is detailed in Law 12 of the Laws of the Game (FIFA, 2015). In our study, the foul play situations concern physical play, including tackling, pushing, or holding an opponent. The in-game perspective is important as researchers have shown that expertise-based differences become more evident as the task and experimental design are representative and closely aligned with the demands of the sport context (Dicks, Davids, & Button, 2009a; Put et al., 2014; Roca, Williams, & Ford, 2013b).

**Fig. 1:**
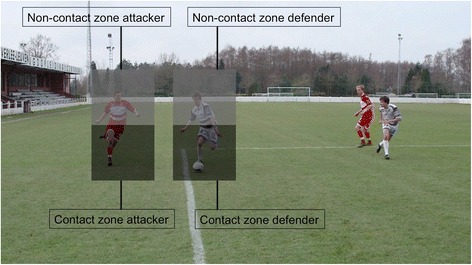
Example of an open play situation with a visualization of the different areas of interest

**Fig. 2:**
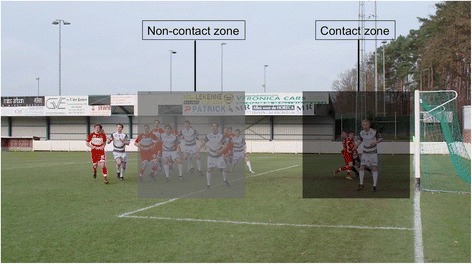
Example of a corner kick situation with a visualization of the different areas of interest

A selection of competitive football players, aged between 19 and 21 years, simulated the foul play situations, which occurred at a distance approximately 10 meters away from the position of the camera. Prior to the start of the filming session, a one-hour practice session was provided in which the players were instructed about the different types of situations and possible infringements. In order to have the situations acted out as naturalistically as possible, however, no specific instructions related to the type of infringement that should be executed were given to either the attacker or defender during the actual play. The attacking and defending teams were clearly distinguished in every situation as one team wore red and the other team wore white shirts, shorts, and socks. The situations took place inside and around the penalty area and there were two different types of situations: open play situations and corner kick situations. These situations varied in the number of players presented and the way the situations were started. During the open play situations, one or two attackers played against two defenders (1 versus 2 or 2 versus 2). The ball was brought into play and it was clear that a possible infringement (e.g., tackle or push) occurred between two players (Fig. 1; Additional file 1). The video clips of the corner kick situations started when a player kicked the ball from the corner arc. Subsequently, 13–14 players (6–7 attackers versus 6–7 defenders and 1 goalkeeper) were involved in front of the goal and two players from this group interacted for a possible infringement (Fig. 2; Additional file 2). Each video clip contained only one potential interaction or infringement.

In cooperation with the Refereeing Officers of the Union des Associations Européennes de Football (UEFA), 20 appropriate video clips (i.e., ten open play situations and ten corner kick situations) were selected out of a total of 90 situations. All situations were representative of regular foul play in professional football: in ten situations the defender tackled the legs of the attacker; in ten situations the defender pushed or held the attacker. Altogether, 17 out of 20 situations resulted in an actual infringement of the Laws of the Game. The video clips of both the open play and corner kick situations lasted approximately 7 s (range 5–9 s).

### Apparatus

Video clips were presented on a Tobii T120 Eye Tracking 17-inch monitor (Tobii Technology AB, Sweden) with a screen resolution of 1280 × 1024 pixels. This system records visual search behavior at a constant frame rate of 120 Hz using five infrared lights and a camera. Measures of visual search behavior (*search rate* and *fixation location*; see the “Dependent variables and data analysis” section below) were calculated using the Tobii Studio version 3.2.1 software.

### Procedure

Before the start of the test session, referees filled out a questionnaire regarding their general experience as a referee. The referees were seated at a distance of 60 cm from the Tobii monitor and a five-point eye calibration was performed. Participants were provided with a standardized explanation of the test procedure and one video clip was used to help familiarize themselves with the test. During the actual test, a video clip of a corner kick situation was always followed by an open play situation. Participants were asked to indicate their decisions for each video clip according to the Laws of the Game of FIFA as they would normally do during a match (FIFA, 2015). First, participants had to make the appropriate technical decision out of four options: no foul; indirect free kick; direct free kick; or penalty kick. Second, participants had to make the appropriate disciplinary decision out of three caution options: no card; yellow card; or red card. Participants received no feedback about their performance and gaze behavior was recorded during completion of the 20 video clips. The entire test session lasted approximately 30 minutes.

### Dependent variables and data analysis

#### Visual search behavior

A fixation was defined as the period of time when the eyes remained stationary within 1° of movement tolerance for a period equal to, or greater than, 120 ms (Catteeuw et al., 2009). Two measures of visual search behavior were recorded for each video clip.

#### Search rate

Search rate refers to the mean number of fixations and the mean fixation duration (in seconds) per trial, irrespective of the location of the fixations.

#### Fixation location

The average total fixation time was calculated during the most crucial part of the video clips (2 s), that is, from the onset of the possible infringement (1 s pre-contact) until the end of the interaction between the players on which the decision is based (1 s post-contact). Average total fixation time refers to the amount of time referees spent fixating various areas of interest within the display prior to making a decision. For the open play situations, a distinction was made between the attacking and defending player. For both players a contact zone (i.e., the body part which was involved in the possible infringement), and a non-contact zone (i.e., the body part which was not involved in the possible infringement) were defined, resulting in a total of four dynamic areas of interest (100 × 150 pixels) (Fig. 1). The contact zones of the attacker and defender partly overlap during the time period surrounding the moment of contact. A fixation could thus be counted in two areas of interest and the average total fixation times could sum to more than 2 s. For the corner kick situations, two dynamic areas of interest (300 × 300 pixels) were used: a contact zone (i.e., the area of the display which contained at least the two players who interacted for the possible infringement); and a non-contact zone (i.e., the area of the display which contained at least two players who were not involved during the possible infringement) (Fig. 2). The size of the dynamic areas of interest was kept constant within and across video clips.

#### Decision-making accuracy

Three independent and experienced ex-FIFA referees selected the correct technical and disciplinary decision for each situation based on the regulations regarding the application of Law 12: Fouls and Misconduct (FIFA, 2015). Only video clips for which all three experts reached a consensus were included. The accuracy scores for the technical and disciplinary decision were calculated separately as the total number of correct trials (in percentage), that is, decisions that were in correspondence with the reference decision.

#### Types of errors

We explored the eye-position data to identify the possible causes of errors for situations where either the technical or disciplinary decision was incorrect. For these situations, a perception error was present when the total fixation time in the contact zone was lower than 1000 ms, which is considered a significant allocation of visual attention (Hillstrom, 2000; Nodine et al., 2002). When the total fixation time in the contact zone was equal to or exceeded 1000 ms, the error was probably due to a failure to categorize the situation according to the Laws of the Game (categorization error). For the open play situations, the total fixation times in the contact zone of the attacking player were taken into account.

#### Data analysis

All dependent variables with respect to the visual search behavior were analyzed separately for open play situations and corner kick situations. Independent t-tests were used to study the differences in search rate (number of fixations and fixation duration) between both groups. Fixation locations for the open play situations were analyzed using a four-way ANOVA with group (elite versus sub-elite) as the between-participants factor and fixated player (attacker versus defender), fixated zone (contact zone versus non-contact zone), and time interval (pre-contact versus post-contact) as the within-participants factors. Fixation locations for the corner kick situations were analyzed using a three-way ANOVA with group (elite versus sub-elite) as the between-participants factor and fixated zone (contact zone versus non-contact zone) and time interval (pre-contact versus post-contact) as the within-participants factors. A three-way ANOVA with group (elite versus sub-elite) as the between-participants factor and situation (open play versus corner kick) and decision (technical versus disciplinary decision) as the within-participants factors was used to study decision-making accuracy. Effect sizes were calculated as partial eta-squared values (η_p_^2^) and Cohen’s d, as appropriate. Partial eta-squared values of 0.01, 0.06, and 0.14 were interpreted as small, medium, or large effects, respectively. Cohen’s d values for small, medium, and large effects are 0.20, 0.50, and 0.80, respectively (Cohen, 1988). A *P* value of <0.05 was considered significant (Field, 2005). Tukey’s HSD post hoc tests were used to follow up interaction effects. All statistical analyses were carried out using IBM SPSS statistical program version 22.

## Results

### Decision-making accuracy

Mean accuracy scores across groups are presented in Table 2. A significant main effect of situation was observed [F(1,37) = 46.172, *P* < 0.001, *η*_*p*_^*2*^ = 0.555]; the accuracy scores for the open play situations (mean (M) = 52.6, SE = 2.54) were significantly lower compared with the corner kick situations (M = 72.9, SE = 1.12). The significant main effect of group indicated that elite referees were overall more accurate than sub-elite referees [F(1,37) = 10.729, *P* = 0.002, *η*_*p*_^*2*^ = 0.225]. There was also a main effect of decision [F(1,37) = 39.639, *P* < 0.001, *η*_*p*_^*2*^ = 0.517], a significant situation × decision interaction effect [F(1,37) = 31.077, *P* < 0.001, *η*_*p*_^*2*^ = 0.456], and a group × situation × decision interaction effect [F(1,37) = 12.882, *P* = 0.001, *η*_*p*_^*2*^ = 0.258]. For the determination of the disciplinary decision in open play situations, the group of elite referees (M = 61.0%, SE = 3.76) scored significantly higher compared with the group of sub-elite referees (M = 45.3%, SE = 4.74). In these open play situations, no differences were found between both groups for the determination of the technical decision. For the determination of the technical decision in corner kick situations, the accuracy scores of the group of elite referees (M = 69.5%, SE = 2.85) were significantly higher compared with the group of sub-elite referees (M = 56.8%, SE = 2.42). In these corner kick situations, no differences were found between both groups for the determination of the disciplinary decision. No significant interaction effects of group × situation [F(1,37) = 0.475, *P* = 0.495, *η*_*p*_^*2*^ = 0.013] and group × decision [F(1,37) = 0.102, *P* = 0.752, *η*_*p*_^*2*^ = 0.003] were observed.

**Table 2:** Mean accuracy scores in percentage (and standard errors) for the elite and sub-elite groups of referees

	Foul/no foul accuracy			
	Open play		Corner kick	
	Technical	Disciplinary	Technical	Disciplinary
Elite	54.5 (4.3)	61.0 (3.8)	69.5 (2.9)	82.5 (1.2)
Sub-elite	49.5 (3.1)	45.3 (4.7)	56.8 (2.4)	82.6 (1.7)

*Notes*: *Technical* represents technical decision; *Disciplinary* represents disciplinary decision

### Visual search behavior

#### Open play situations

##### Search rate

No significant differences in the total number of fixations were observed between groups (t(1,37) = −0.428, *P* = 0.671, d = 0.136). Moreover, the mean fixation duration did not differ across groups (t(1,37) = 0.179, *P* = 0.859, d = 0.057) (Table 3).

**Table 3:** Mean number of fixations (and standard errors) and mean fixation durations (and standard errors) for the elite and sub-elite group of referees

	Open play		Corner kick	
	Elite	Sub-elite	Elite	Sub-elite
Number of fixations	16.9 (0.6)	17.2 (0.6)	19.1 (0.5)	19.6 (0.6)
Mean fixation duration (s)	0.40 (0.02)	0.40 (0.02)	0.32 (0.01)	0.32 (0.02)

##### Fixation location

**Fig. 3:**
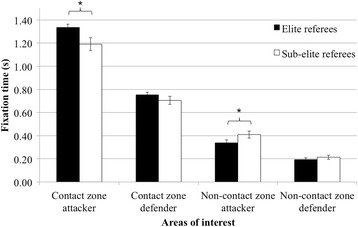
Average total fixation times (and standard errors) in each area of interest across the open play situations for the elite and sub-elite group of referees. *P < 0.05

The average total fixation times in each area of interest and for each group are presented in Fig. 3. The analysis revealed a significant main effect of the fixated player [F(1,37) = 495.201, *P* < 0.001, *η*_*p*_^*2*^ = 0.930]. Referees spent significantly more time fixating the attacking player (M = 1.64 s, SE = 0.03) in comparison with the defending player (M = 0.93 s, SE = 0.02). A significant main effect of fixated zone was found [F(1,37) = 653.722, *P* < 0.001, *η*_*p*_^*2*^ = 0.946]. There was a significant group × fixated zone interaction effect [F(1,37) = 6.469, *P* = 0.015, *η*_*p*_^*2*^ = 0.149] and a fixated player × fixated zone interaction effect [F(1,37) = 110.131, *P* < 0.001, *η*_*p*_^*2*^ = 0.749], but no main effect of group [F(1,37) = 1.182, *P* = 0.284, *η*_*p*_^*2*^ = 0.031] and no group × fixated player interaction effect [F(1,37) = 0.449, *P* = 0.507, *η*_*p*_^*2*^ = 0.012]. Furthermore, a significant group × fixated player × fixated zone interaction effect was observed [F(1,37) = 4.497, *P* = 0.041, *η*_*p*_^*2*^ = 0.108]. Post hoc testing showed that referees in the elite group spent more time fixating the contact zone of the attacker (M = 1.33 s, SE = 0.03) and less time fixating the non-contact zone of the attacker (M = 0.34 s, SE = 0.02) compared with the sub-elite referees (M = 1.19 s, SE = 0.06 and M = 0.41 s, SE = 0.03, respectively). No significant differences were observed between both groups for the time spent fixating the contact zone and non-contact zone of the defender. There was a main effect of time interval [F(1,37) = 467.747, *P* < 0.001, *η*_*p*_^*2*^ = 0.927], a significant time interval × fixated player interaction effect [F(1,37) = 12.149, *P* = 0.001, *η*_*p*_^*2*^ = 0.248], a time interval × fixated zone interaction effect [F(1,37) = 23.933, *P* < 0.001, *η*_*p*_^*2*^ = 0.393], and a time interval × fixated player × fixated zone interaction effect [F(1,37) = 154.143, *P* < 0.001, *η*_*p*_^*2*^ = 0.806]. Importantly, these time interval effects did not interact with the between-participants factor group.

#### Corner kick situations

##### Search rate

No significant differences in the total number of fixations were observed between groups (t(1,37) = −0.647, *P* = 0.522, d = 0.208). The mean fixation duration did not differ significantly (t(1,37) = 0.156, *P* = 0.877, d = 0.005) (Table 3).

##### Fixation location

**Fig. 4:**
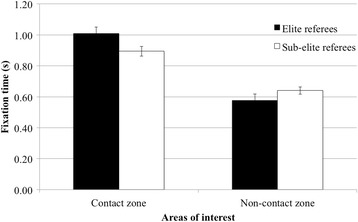
Average total fixation times (and standard errors) in each area of interest across the corner kick situations for the elite and sub-elite group of referees

The average total fixation times in each area of interest across groups are presented in Fig. 4. A significant main effect of fixated zone was registered [F(1,37) = 51.653, *P* < 0.001, *η*_*p*_^*2*^ = 0.583]. Referees spent significantly more time fixating the contact zone (M = 0.95 s, SE = 0.03) in comparison with the non-contact zone (M = 0.61, SE = 0.02). There was no main effect of group [F(1,37) = 1.857, *P* = 0.181, *η*_*p*_^*2*^ = 0.048]. The group × fixated zone interaction effect approaches significance [F(1,37) = 3.457, *P* = 0.071, *η*_*p*_^*2*^ = 0.085], indicating that the average total fixation time in the contact zone and non-contact zone tend to diverge between the elite and sub-elite referees (Fig. 4). Furthermore, a significant main effect of time interval [F(1,37) = 26.063, *P* < 0.001, *η*_*p*_^*2*^ = 0.413] and a time interval × fixated zone interaction effect [F(1,37) = 66.261, *P* < 0.001, *η*_*p*_^*2*^ = 0.642] were observed. Importantly, the time interval effects did not interact with the between-participants factor group.

### Types of errors

If an incorrect decision was given for a situation (incorrect technical and/or disciplinary decision), this could be the result of an error in the perception or the categorization process. An overview of the different types of errors, based on the total fixation times in the contact zone, is presented in Table 4. Situations for which both the technical and disciplinary decision was correct were categorized under “correct perception and categorization”.

**Table 4:** Classification of types of errors (percentages of situations) for both groups of referees based on the total fixation times

	Open play		Corner kick	
	Elite	Sub-elite	Elite	Sub-elite
Perception error	12%	18%	20%	26%
Categorization error	42%	46%	20%	22%
Correct perception and categorization	46%	36%	60%	52%

*Notes*: *Perception error* represents total fixation time contact zone <1000 ms; *Categorization error* represents total fixation time contact zone ≥1000 ms

## Discussion

Although refereeing is a complex domain of expertise requiring a variety of different perceptual-cognitive skills, scientists have paid limited attention to the decision-making processes involved (MacMahon et al., 2014). In this study, we examined differences in the visual search behavior and decision-making accuracy between elite and sub-elite referees. We predicted, based on previous research involving the analysis of referees’ decisions, that there would be systematic expertise-based differences in the decision-making accuracy (Catteeuw et al., 2009; Gilis et al., 2008; Hancock & Ste-Marie, 2013; Mascarenhas et al., 2005). For both the open play and corner kick situations, our results showed that the decisions of the elite group were more accurate than the sub-elite group. We suggest that elite referees have developed specific and elaborate knowledge structures, which can be used to make appropriate decisions in a dynamic time-constrained environment (LTWM; Ericsson & Kintsch, 1995). The performance of the elite referees in this study was in line with previous research reporting the decision-making accuracy of elite association football referees. During crucial incidents in real games the range of reported accuracy is between 64 and 77% (Dicks, O’Hare, Button, & Mascarenhas, 2009b; Gilis, Weston, Helsen, Junge, & Dvorak, 2006).

We were mainly interested in the visual search strategies that were adopted by both groups of referees in an effort to identify the mediating mechanisms underlying the superior performance of the elite group of referees over the sub-elite group (Williams & Ericsson, 2005). Previously, researchers have shown that visual search strategies depend on the specific domain of expertise and task demands. Thus far, only two published reports have registered the eye movements of referees during a decision-making task (Catteeuw et al., 2009; Hancock & Ste-Marie, 2013). In the present study, we examined whether elite referees employ a more effective scan pattern in comparison with referees of a sub-elite level.

No differences were reported in search rate across groups. These results are in accordance with previous research within the domain of refereeing, showing no differences in the number and duration of the fixations. It was argued that differences in the decision-making process between referees are possibly due to the ability to extract better quality information per fixation and the ability to acquire information more effectively via peripheral vision (Catteeuw et al., 2009; Hancock & Ste-Marie, 2013). The variables used to measure visual search rate, that is, the total number of fixations and the fixation duration, are indicative of the amount of information processed by the referee.

The precise location of the fixation is another important variable and indicates the area of interest where referees fixate. This study was the first to investigate the allocation of point of gaze during a foul play assessment task. We were particularly interested in whether there were differences in the way elite and sub-elite referees use specific areas of interest in order to guide subsequent decision making. The time spent fixating specific areas of interest was analyzed during the most critical part of the video clip; that is, from the moment a defender started to interfere with an opponent (1 s pre-contact) until the end of the interaction between both players (1 s post-contact). For the open play situations, referees in the elite group spent more time fixating the most informative area of the attacking player and less time fixating the body part that was not involved in the infringement compared with the sub-elite group. Furthermore, for the corner kick situations the interaction between group and fixated zone approached significance. Although this result should be approached cautiously, it suggests that, for the corner kick situations, the group of elite referees spent more time fixating the contact zone and less time fixating the non-contact zone compared with the sub-elite referees. This study is the first to show differences between elite and sub-elite referees with respect to the fixated area of the display. We speculate that elite referees illustrate a tendency to identify, focus, and interpret the most crucial information within the visual display (i.e., the contact zones). The sub-elite referees tended to rely on less relevant information, spending longer periods of time fixating on the non-contact zones, preventing them from generating accurate and complete mental representations of the situation (Ericsson & Kintsch, 1995).

Although the results of the current study may be to some extent context or task specific, our general approach might still have practical utility in a variety of professional settings. The rich, complex situations which officials encounter are representative of many of the challenging tasks that experts carry out in their professional lives. Therefore, our findings may have implications for the development of realistic perceptual-cognitive tasks and test protocols studying the underlying mechanisms of expertise across domains. In particular, the difficulty of referees in association football is that the physical contacts between the players must be judged according to how careless, reckless, or excessively forceful they are. This means that an important categorical decision component is added to the process after the relevant areas of the clips are selected and perceived correctly. So, in addition to selection and perception, categorization and decision-making processes are involved, which makes this a highly demanding task to be carried out under time pressure. It is not clear a priori to what extent expertise in this domain is due to any or all of these processes. Our eye movement study helps to clarify some of this dynamic interplay. We hope that our study will provide inspiration as well as methodological and empirical foundation for other areas of expertise in which time-constraint decision-making in a complex environment is key.

Previously, researchers have focused on the biases that occur during encoding/retrieving information and the impact of various external and contextual factors (e.g., previous decisions, crowd noise) on the decision-making process of referees (Balmer et al., 2007; Plessner & Betsch, 2001; Unkelbach & Memmert, 2010). However, a situation must first be perceived accurately, so that the relevant information can be brought into the processing system (Plessner & Haar, 2006). The influence of perceptual processes on judgment and decision making by sports officials is evident in a number of studies, showing that an incorrect visual perspective of the referee might bias their decision-making process (Bar-Eli, Plessner, & Raab, 2011; Oudejans et al., 2000). Our study demonstrated that, independent of the viewing perspective, differences occur in the way open play situations are perceived by referees of different levels. We speculate that based on previous experiences with open play situations, experts have acquired more elaborate representations stored in long-term memory and have learned to position their gaze accordingly. As such, their visual search is primarily under top-down control and driven by acquired knowledge. An internal map is formed over which saccades are planned and most informative fixation locations are selected. Sub-elite referees have acquired less experience and still have to learn to pay attention to the key sources of information (MacMahon et al., 2014). They seem to apply a more random or stimulus-driven control of fixation locations (bottom-up), as the representations stored in long-term memory are not strong enough to guide in an efficient way their visual attention. Visual search of sub-elite referees is more dependent on the information that is available in the video clips and, therefore, they are often misled by salient and irrelevant information (Einhäuser, Rutishauser, & Koch, 2008; Malcolm & Henderson, 2010; Tatler, Hayhoe, Land, & Ballard, 2011).

Referees must learn what to look at and when. Accumulated deliberate practice is a prerequisite for the development of LTWM skills and complex retrieval structures. These skills allow referees to control key aspects of the decision-making process and maintain access to task-relevant information, allocating (visual) attention to the informative features of a specific situation. The amount of experience and acquired knowledge structures, however, may vary as a function of the type of situation. Previous research (Helsen & Bultynck, 2004) has shown there are approximately ten corner kick situations during a game. During most of these corner kicks, a limited number of infringements occur, possibly due to a pro-active style of refereeing. On the other hand, a referee has to make approximately 45 technical decisions throughout a match and most of these decisions are made during open play situations. The lack of exposure to corner kick situations might make it difficult to develop strong representations and this could be a reason that no significant differences were found in the visual search data for these situations.

An alternative explanation could be that, for the corner kick situations, there is no single area that one should look at until the moment of contact. Any couple of players could be the contact zone during a corner kick situation and the experts would only be able to use their knowledge to the fullest advantage during the post-contact time interval (under the assumption that they can detect more quickly where the critical event occurred). Our results show that there is no significant difference in the average total fixation time in the contact zone between elite and sub-elite referees during the post-contact time interval of corner kick situations. Therefore, this explanation cannot be confirmed and needs further investigation. During the open play situations, elite referees were able to select and fixate the most relevant information during both the pre- and post-contact time interval.

An error in the decision-making process was attributed to either an error in perception or categorization. A criterion of 1000 ms was used to indicate whether the most crucial area was clearly fixated and perceived (Hillstrom, 2000; Nodine et al., 2002). The classification of errors revealed that, despite proper perception of the situation, a considerable part of erroneous decisions is probably the result of inaccurate categorization. For the elite referees, this was the case in 42% of the open play situations and 20% of the corner kick situations. For the sub-elite referees, this was the case in 46% of the open play situations and 22% of the corner kick situations. Although one can argue that the criterion of 1000 ms is arbitrary, we believe this to be a reasonable estimate of the proportion of categorization errors, showing that it is important to encode and give meaning to the perceived stimulus according to the Laws of the Game.

Finally, a number of limitations and recommendations for future work and training applications are highlighted. Although we presented representative in-game decision-making situations, there might still be expertise-based differences between these film-based situations and in situ situations (Dicks, Button, & Davids, 2010). Our video clips were filmed from the first-person perspective of the additional assistant referee and by collecting the visual search data in a laboratory setting, we were able to maintain experimental control and recreate standardized and reproducible conditions needed for comparisons between and within groups. As such, we were able to address a real-world application of psychological principles. A similar protocol can be used to objectively and reproducibly test performance and expertise effects across different domains. In future research, however, it could be a good idea to collect visual search data in live-action settings using a portable eye-tracking system, to further enhance the ecological validity of the research.

In future, researchers should focus on the development of effective training methods. There is a continuous need for improving the refereeing standards and the present data have important implications for those involved in developing such training programs within the domain of refereeing. Until now, training has mainly focused on the physical component and the decision-making skills are often only trained during actual match situations. However, video-based training programs can be effective in offering additional refereeing experience (Catteeuw, Gilis, Jaspers, Wagemans, & Helsen, 2010; Put et al., 2015; Schweizer et al., 2011; Schweizer, Plessner, & Brand, 2013). Training programs can be designed to teach promising referees to look, think, and act like elite referees and immunize them against the undesired influence of irrelevant cues. Looking in the right place at the right time is important in refereeing, but definitely also in other sports and professional settings, such as airport baggage screening, medical screening, and law enforcement. A better understanding of the underlying mechanisms of expertise is fundamental to guide and improve the decision-making process across domains. By implicitly directing attention to the crucial features via guided discovery, for example, we might facilitate the development of more effective visual search behaviors (Savelsbergh, van Gastel, & van Kampen, 2010). Providing relevant feedback and creating a “kind-learning environment” are important features of the training setting, which should be used to emphasize and fine-tune the probabilistic relationship between observable cues and the decision-making process (Hogarth, 2008). The findings of this study have significant implications for the manner in which researchers and those involved in the training process try to capture and develop perceptual-cognitive skills in sports and other domains.

## Conclusions

This study was the first to explore the decision-making skills and visual search strategies that were adopted by a group of elite and sub-elite association football referees while assessing foul play situations. Elite referees made more accurate decisions and there were systematic differences in their visual search behaviors. These differences in the information selection for perceptual and cognitive processing may have practical utility for the testing and training of sports officials and experts across other domains as the presented situations are representative of many of the challenging tasks that experts carry out in their professional lives.

## Additional files

Additional file 1: Video clip of an open play situation (AVI 27544 kb)

Additional file 2: Video clip of a corner kick situation (AVI 27179 kb)
